# Acyl homoserine lactone-mediated quorum sensing in the oral cavity: a paradigm revisited

**DOI:** 10.1038/s41598-020-66704-4

**Published:** 2020-06-17

**Authors:** Andrea Muras, Paz Otero-Casal, Vanessa Blanc, Ana Otero

**Affiliations:** 1 Departamento de Microbioloxía e Parasitoloxía, Facultade de Bioloxía-CIBUS, Universidade de Santiago de Compostela, Santiago de Compostela, Spain; 2 Departamento de Ciruxía e Especialidade Médico-Cirúrxica, Facultade de Medicina e Odontoloxía, Universidade de Santiago de Compostela, Santiago de Compostela, Spain; 3 Unit of Oral Health, C.S. Santa Comba-Negreira, SERGAS, Spain; 4 Department of Microbiology, Dentaid Research Center, Dentaid S.L., Barcelona, Spain

**Keywords:** Microbiology, Molecular biology

## Abstract

Acyl homoserine lactones (AHLs), the quorum sensing (QS) signals produced by Gram-negative bacteria, are currently considered to play a minor role in the development of oral biofilm since their production by oral pathogens has not been ascertained thus far. However, we report the presence of AHLs in different oral samples and their production by the oral pathogen *Porphyromonas gingivalis*. The importance of AHLs is further supported by a very high prevalence of AHL-degradation capability, up to 60%, among bacteria isolated from dental plaque and saliva samples. Furthermore, the wide-spectrum AHL-lactonase Aii20J significantly inhibited oral biofilm formation in different *in vitro* biofilm models and caused important changes in bacterial composition. Besides, the inhibitory effect of Aii20J on a mixed biofilm of 6 oral pathogens was verified using confocal microscopy. Much more research is needed in order to be able to associate specific AHLs with oral pathologies and to individuate the key actors in AHL-mediated QS processes in dental plaque formation. However, these results indicate a higher relevance of the AHLs in the oral cavity than generally accepted thus far and suggest the potential use of inhibitory strategies against these signals for the prevention and treatment of oral diseases.

## Introduction

Dental plaque is an extremely complex biofilm that results from the accumulation and interaction of oral microorganisms attached to the tooth surface, embedded in a matrix of extracellular polymers. During dental plaque development, the ecological equilibrium of the microbes in the oral cavity is maintained through competitive and cooperative interactions. Indeed, an imbalance of the resident microbiota derived from a change in local environmental conditions is responsible for the two major oral bacterial diseases: caries and periodontal diseases^1–3^. The responsibility for the progression of these oral diseases cannot be attributed to a single oral pathogen, instead the entire community resident in the oral cavity, as well as its functional activities, are responsible for their development^4,5^. Furthermore, other important pathologies, including Alzheimer’s disease and oral cancer, are also thought to be related to oral bacteria^6,7^. In this context, the interference with the oral biofilm formation processes, through the inhibition of bacterial coaggregation and/or the reduction of dental plaque formation is supposed to have beneficial effects on oral health^8^.

Some of the potential targets for the development of strategies to inhibit dental plaque formation are the bacterial communication processes known as Quorum sensing (QS). These cell density-dependent mechanisms which control gene expression are accepted as essential for the successful establishment of bacterial biofilms. However, the current knowledge about signalling processes and microbial interactions within oral biofilms is still limited, and their effects on commensal microbiota and the establishment of dysbiosis remain unclear^9^. The QS molecules produced by Gram-positive bacteria, the AutoInducer Peptides (AIPs), have been identified in different oral streptococci. The production of the Autoinductor-2 (AI-2), signals an interconvertible group of signalling molecules based on a furanosyl borate diester, was also detected in different Gram-positive and Gram-negative oral pathogenic bacteria such as *Streptococcus mutans* or *Porphyromonas gingivalis*^10–13^. Among the different QS signal molecules described so far, the N-acyl homoserine lactones (AHLs), molecules constituted by a homoserine lactone ring (HSL) linked by an amide bond to a fatty acid (between 4 and 20 carbons), are the best studied and characterized. The specificity of these molecules is dependent on the length of the molecule and presence of hydroxy- or oxo- substitutions in the third carbon. However, attempts to detect the production of the AHL signals, typical of Gram-negative bacteria, by oral pathogens have remained unsuccessful^10–12^. Therefore, AHLs have not been assigned an important role in microbe-microbe interactions within dental plaque^14–16^. Despite that, an increasing number of direct and indirect evidence has been accumulated in recent years, pointing to AHLs’ role in dental plaque formation. AHLs have been detected in saliva and sputum samples^17–20^. Additionally, several AHL-producing strains of *Enterobacter* sp., *Klebsiella pneumoniae*, *Pseudomonas putida, Citrobacter amalonaticus* (*Levinea amalonaticus*) L8A and *Burkholderia* sp. have been isolated from the human tongue surface and dental plaque samples^21–25^. Furthermore, a homologue of the AHL-synthase HdtS, as well as a LuxR-type receptor homologue, were identified in *P. gingivalis* W83 and *P. gingivalis* ATCC33277, respectively^26–28^. In this context, previous studies observed that AHLs and AHL-analogues modified not only the protein expression but also slowed down the growth in *P. gingivalis*^26,29^. Recently, we have also demonstrated that the exogenous addition of specific AHLs affects pathology-related phenotypes such as lactic acid production and protease activity in *in vitro* oral biofilm models^19^. In these models, *N*-hexanoyl-L-homoserine lactone (C6-HSL) increases the relative presence of *Peptostreptococcus* and *Prevotella*, producing a shift towards a periodontal bacterial composition profile^19^. Altogether, these results point to a possible role of AHL-mediated QS in the oral cavity. The confirmation of the critical function of AHLs in oral biofilm formation would open new opportunities in the prevention and treatment of oral infectious diseases since the most active QS inhibitors described thus far interfere with AHL-mediated QS systems.

Numerous organisms have evolved the capacity to inhibit QS systems, a process generally known as Quorum Sensing Inhibition (QSI) or Quorum Quenching (QQ), probably because these cell-to-cell communication systems play a key role in the interactions, not only between prokaryotes but also with eukaryotes. Enzymatic QQ is the best-studied QS inhibitory strategy^30^. The genes that codify this type of enzymes are classified in two main groups: lactonases and acylases, although other types of QQ enzymes have also been described^31^. The interference with QS processes has become an interesting alternative for fighting the problem of bacterial antibiotic resistance and has been proposed as a promising approach for controlling different pathogenic bacterial traits in order to prevent or treat infectious diseases^31–35^. Furthermore, the use of QQ compounds also increases biofilm-forming pathogens´ susceptibility to antibiotics^36^. Since QQ strategies do not interfere directly with bacterial growth, the probabilities of inducing tolerance or resistance against these mechanisms are lower^37,38^. Previous studies have already reported the successful approach of using QS inhibitors to control different types of bacterial biofilms^35,39–42^. Therefore, the confirmation of a possible role of AHL-type QS signals in dental plaque formation would open new perspectives in the prevention and treatment of oral diseases.

This study describes the presence of AHLs in oral samples (saliva and extracted teeth) and their production by *P. gingivalis* indicating that this type of QS signal plays a potential role in the establishment of the oral microbial communities. Furthermore, in order to evaluate the importance of these QS signals in the process of oral biofilm formation, the effect of the wide-spectrum, thermostable AHL-lactonase Aii20J^33^, obtained from the marine bacterium *Tenacibaculum* sp. 20J^43^, was tested on different *in vitro* oral biofilms obtained from saliva samples from healthy and unhealthy donors. Important inhibition was observed using the xCELLigence monitoring system, which allows real-time measurements of surface-associated bacterial growth^35,44^ and a modification of the Amsterdam Active Attachment biofilm model^19,45^. In addition, the inhibitory effect of the QQ enzyme Aii20J was also observed on *in vitro* multi-species biofilms formed by six oral pathogens. All these data strongly support the important role AHLs play in oral biofilm formation. However, much more research is necessary in order to be able to associate AHLs with oral pathologies and to individuate the key actors in AHL-mediated QS processes in dental plaque formation.

## Results

### AHL-type quorum sensing signals detection in oral samples and mixed biofilm

**Figure 1:**
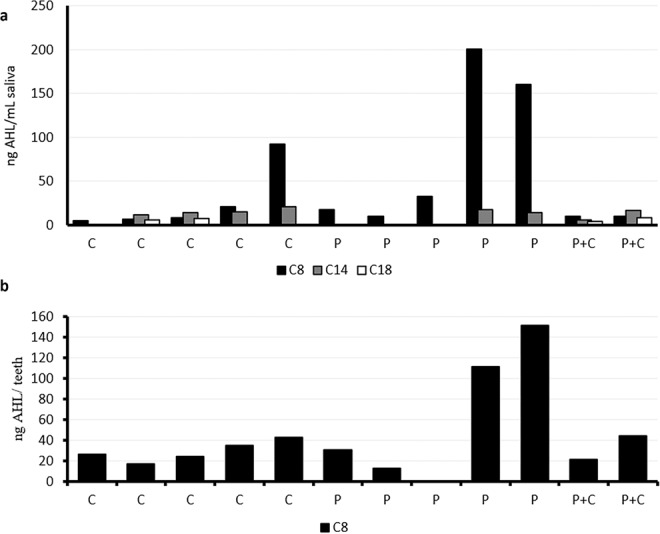
Detection of acyl homoserine lactones (AHLs) in saliva samples (**a**) and extracted teeth (**b**) obtained from the same patients using HPLC-MS. P = periodontal disease; C = caries.

The presence of AHL-type QS signals was evaluated in two different types of oral samples from the same patient: extracted teeth and saliva samples. The analysis of saliva obtained from different patients unequivocally demonstrated the presence of three AHLs (Supplementary material Figs. 1, 2 and 3): *N*-octanoyl-L-homoserine lactone (C8-HSL), *N*-tetradecanoyl-L-homoserine lactone (C14-HSL) and *N*-octadecanoyl-L-homoserine lactone (C18-HSL) (Fig. 1a). The QS molecule C8-HSL was not only the most abundant AHL (4.97–200.27 ng/mL), but it was also present in all the saliva samples. Those saliva samples in which the signals C8, C14, and C18-HSL were present were obtained from patients with caries lesions while only C8-HSL and occasionally C14-HSL were found in saliva from periodontal patients. Additionally, C8-HSL was also detected in most of the extracted teeth (Fig. 1b).

The analysis of the supernatant obtained from a mixed biolfilm culture formed by the six bacterial pathogens *S. oralis, Veillonella parvula, Actinomyces naeslundii, Fusobacterium nucleatum, Aggregatibacter actinomycetemcomitans* and *P. gingivalis* revealed the presence of the QS signal *N*-oxo-octanoyl-L-homoserine lactone (OC8-HSL) (0.87 ng/mL). In order to know if *P. gingivalis* was the strain responsible for the AHL production, this bacterium was cultured axenically and co-cultured with the Gram-positives *S. gordonii* or *S. oralis*. The data showed that a monospecific culture of *P. gingivalis* produced a small quantity of OC8-HSL (0.30 ng/mL), but a higher amount of this AHL was observed when this oral pathogen was cultured in a dual-species biofilm with *S. gordonii* (0.83 ng/mL) or *S. oralis* (1.4 ng/mL).

### Quorum quenching activity in the oral cavity

As a complementary approach to the analysis of AHLs in oral samples, the presence of QQ activity was also analyzed. A total of 567 bacterial isolates, 295 from a healthy patient and 272 from a periodontal patient, were obtained from saliva and dental plaque samples (Supplementary material Table 1). The capacity of this oral bacterial collection to interfere with the short-chain AHLs was tested using a *Chromobacterium violaceum*-based bioassay^46^. Among the 567 oral isolates, the ability to quench the short-chain QS signal C6-HSL was observed as the complete inhibition of violacein production in a surprisingly large number of cultivable oral bacteria (Supplementary material Table 1). The average activity was higher in the periodontal samples (37.42%) than in the healthy ones (23.20%). This difference was even more evident if only dental plaque isolates were compared, with 46.71% of isolates presenting QQ activity for the periodontal sample, against only 14% of isolates from the healthy donor. Since activity against short-chain AHLs is generally much less frequent than the activity against long-chain AHLs^46^, the total QQ activity in the oral cultivable bacteria may be even higher. The *Agrobacterium tumefaciens* bioassays^46^ did not produce consistent results regarding the production of AHLs in these isolates but revealed that 73 strains had antibiotic activity against this bacterium biosensor: 44 were isolated from the healthy donor (5 from dental plaque and 39 from saliva), and 29 were obtained from the periodontal patient (14 from dental plaque and 15 from saliva). This higher antimicrobial activity in the healthy patient (60.27%) compared to the values of the periodontal one (39.72%) could be related with the health status of the donors, although it should be noted that these results are based on isolates from a single patient. The degradation of C12-HSL was found in almost all the saliva samples analyzed, but C6-HSL was only partially reduced in a few samples (data not shown).

### Effect of the AHL-lactonase Aii20J on *in vitro* oral biofilm formation measured by xCELLigence system

**Figure 2:**
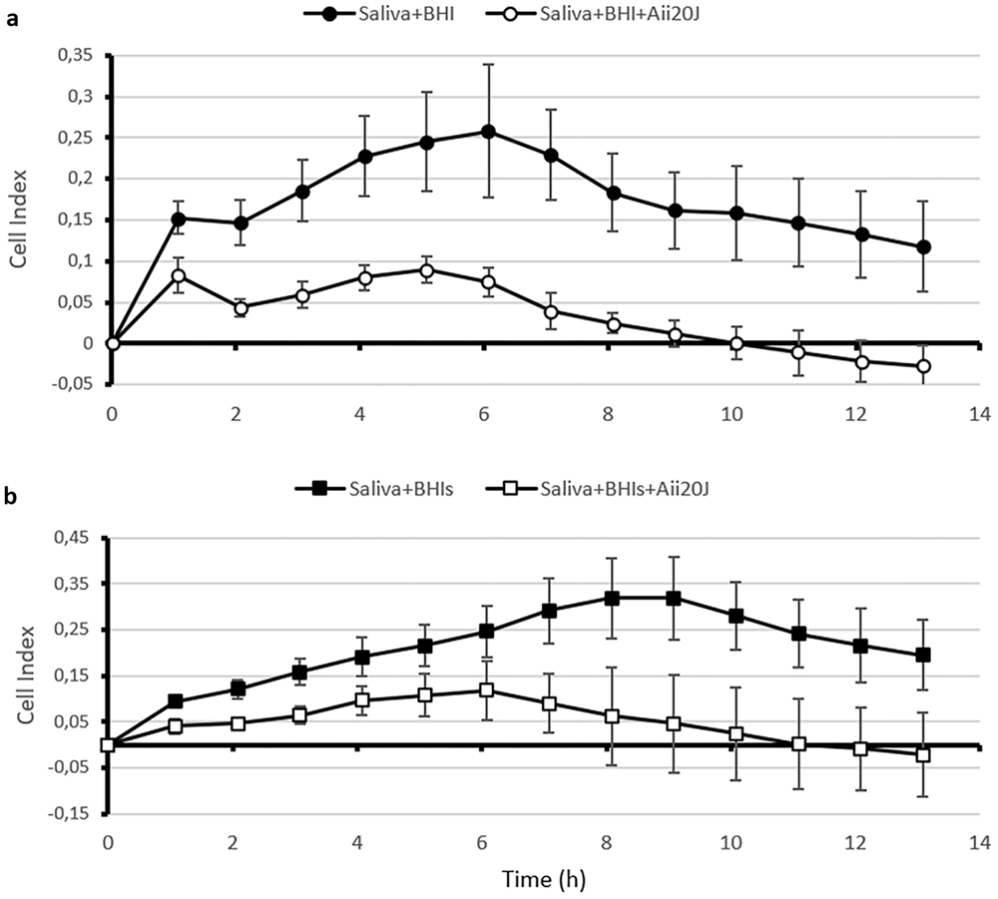
Effect of the AHL-lactonase Aii20J (20 µg/mL) on *in vitro* oral biofilm obtained from the saliva of a healthy donor as measured using the xCELLigence system. The culture was done in BHI (**a**) and BHI supplemented with 0.1% sucrose (BHIs) (**b**). Biofilm formation was expressed in Cell Index units. Data are representative of 3 independent experiments.

Since the presence of different AHLs was unequivocally demonstrated in oral samples, the effect of the wide-spectrum AHL-lactonase Aii20J on biofilm formation from saliva samples obtained from a healthy patient was tested using the real-time measurement equipment xCELLigence (Fig. 2), as a first “black box” approach, to evaluate the importance of these QS signals in oral biofilm formation. The AHL-lactonase Aii20J caused a significant reduction in saliva oral biofilms grown using either BHI (Fig. 2a) or BHI supplemented with sucrose 0.1% (Fig. 2b) as culture media after only one hour of incubation (Student’s t-test, p = 0.007).

**Figure 3:**
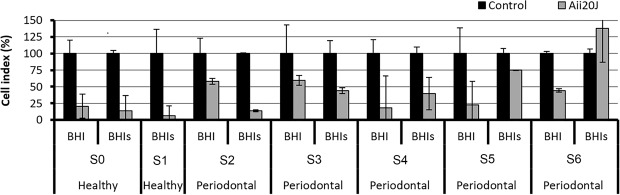
Effect of the AHL-lactonase Aii20J (20 µg/mL) on *in vitro* oral biofilm formed using saliva samples obtained from healthy (S0 and S1), and periodontal patients (S2-S6) in BHI and BHI supplemented with 0.1% sucrose (BHIs) and quantified by the xCELLigence on line measurement system. Measures are shown after 8 h of incubation and expressed as percentage of the control cultures (n = 2).

Given the promising results obtained in the preliminary tests, the capability of Aii20 to inhibit oral biofilms was further evaluated using saliva obtained from other healthy and periodontal patients as inoculum, measured with xCELLigence (Fig. 3). Again, Aii20J caused an important biofilm inhibition (40.47–81.59%) when different saliva samples were inoculated in BHI. In the same way, the biofilm formation in BHI supplemented with 0.1% sucrose (BHIs) showed a high reduction in almost all cases (25.15–93.4%) in the presence of the enzyme. Only one sample (S6) was not affected by Aii20J, probably because the biofilm formed in this case was not strong enough to observe any effect (cell index lower than 0.08, Supplementary material Fig. 4).

### Effect of the AHL-lactonase Aii20J on *in vitro* oral biofilm formation measured by Active Attachment system

In order to avoid the limitations of the xCELLigence system, such as the lack of response in the measurements after 24 h and to allow the possibility of refreshing the culture media, the effect of Aii20J on oral biofilms was tested using a modification of the Amsterdam Active Attachment model^19,45^. This biofilm cultivation model allowed for a higher adhesion surface (up to 1.62 cm^2^) and enabled observation of structural changes in the biofilm. The effect of the refreshment of the culture media on saliva biofilm formation was evaluated using the crystal violet staining, showing a clear increase in the biofilm biomass when culture media was exchanged every 12 h (Supplementary material Fig. 5).

**Figure 4:**
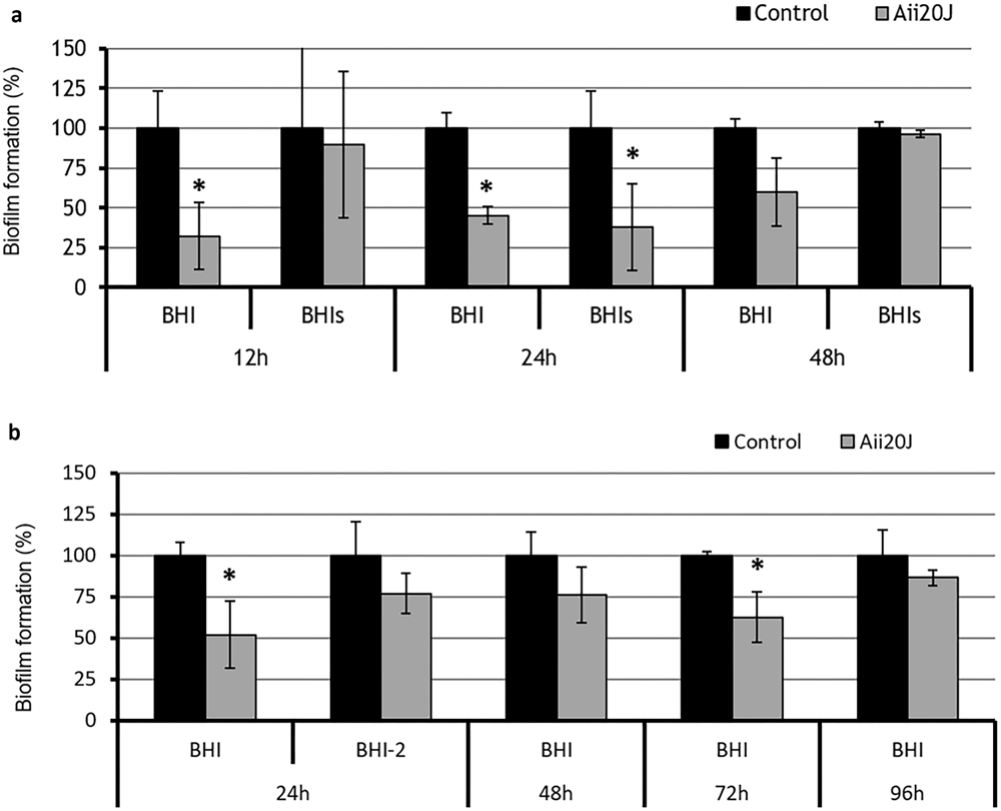
Formation of *in vitro* oral biofilm obtained from saliva samples from a healthy patient with and without the AHL-lactonase Aii20J (20 µg/mL) using the Active Attachment model and measured with the crystal violet staining assay. (**a**) Biofilms incubated in aerobic conditions at 37 °C using BHI and BHI supplemented with 0.1% sucrose (BHIs). Aerobic biofilms were sampled at 24 h, 48 h and 72 h; (**b**) Biofilms incubated in anaerobic conditions at 37 °C using BHI and BHI-2 (BHI supplemented with 2.5 g/L mucin, 1 g/L yeast extract, 0.1 g/L cysteine, 2 g/L sodium bicarbonate, 5 mg/L hemin, 1 mg/L menadione and 0.25% (v/v) glutamic acid). Anaerobic biofilms were sampled at 24 h, 48 h, 72 h and 96 h at 37 °C for BHI. BHI-2 biofilms could only be stained after 24h. Significantly different values are indicated with an asterisk (Student’s t-test, p < 0.05) (n = 3).

The addition of the AHL-lactonase Aii20J (20 µg/mL) caused a significant inhibition of the biofilms formed at 12 h (67.65%) and 24 h (58.14%) in BHI under aerobic conditions (Student’s t-test, p = 0.019, p = 0.003). The inhibitory effect was reduced at 48 h (Fig. 4a). Also, a significant reduction in the biofilm formed in BHI supplemented with sucrose was observed at 24 h of incubation (62.72%) in the presence of the enzyme (Student’s t-test, p = 0.003). The addition of Aii20J in BHI-grown biofilms under anaerobic conditions caused a significant inhibition at 24 h (52.01%) and 72 h (37.41%) (Student’s t-test, p = 0.019, p = 0.04). The measurement of the biofilms using the BHI-2 culture medium (BHI supplemented with 2.5 g/L mucin, 1 g/L yeast extract, 0.1 g/L cysteine, 2 g/L sodium bicarbonate, 5 mg/L hemin, 1 mg/L menadione and 0.25% (v/v) glutamic acid)^47^ in anaerobic conditions could only be done at 24 h (Fig. 4b) since most of the biomass detached from the coverslips obtained at 48 h, 72 h and 92 h during the crystal violet staining procedure (Supplementary material Fig. 6).

Using this biofilm cultivation method, we observed a high inhibitory effect when the AHL-lactonase Aii20J was added to the BHI culture medium using samples from a healthy patient. It was demonstrated that Aii20J caused this anti-biofilm activity since it disappeared when the QQ enzyme was removed by filtration through a 10 kDa membrane and was sharply reduced when the lactonase was autoclaved for 10 minutes (Supplementary material Fig. 7).

The effect of Aii20J (20 µg/mL) on *in vitro* biofilms was further evaluated with several saliva samples obtained from healthy and periodontal patients using BHI as culture media. The biomass obtained in the biofilm formed by the healthy patient used in the previous experiments (saliva 1) was much higher than for other patients (Supplementary material Fig. 8) indicating a high variability in the biofilm formation between individuals. The addition of the enzyme Aii20J reduced biofilm formation in 50% of the samples, with reduction percentages in the range of 20.82–76.44%, although this reduction was statistically significant only for saliva 1 (76.44%) and saliva 8 (26.05%). All the biofilms that reached an optical density value higher than 0.2 in the crystal violet staining assay were affected by the addition of Aii20J.

### Effect of the AHL-lactonase Aii20J on bacterial diversity of *in vitro* oral biofilm

**Figure 5:**
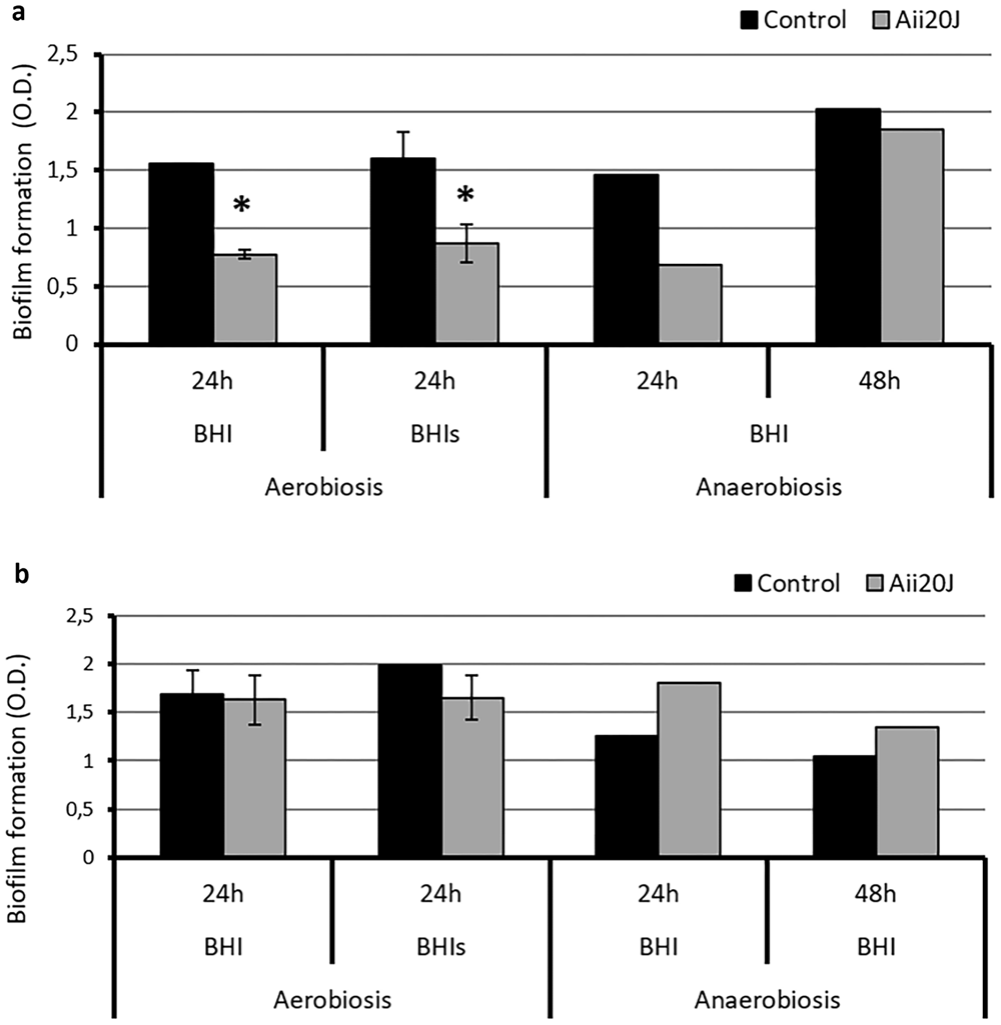
Formation of *in vitro* oral biofilms obtained from saliva samples from a healthy (**a**) and a periodontal (**b**) donor with and without the AHL-lactonase Aii20J (20 µg/mL) using the Active Attachment model and measured with the crystal violet staining assay (O.D. 590 nm). The cultures were done in aerobic conditions using BHI and BHI supplemented with 0.1% sucrose (BHIs) for 24 h (n = 3) and in anaerobic conditions for 24 and 48 h using BHI (n = 1). Significantly different values are indicated with an asterisk (Student’s t-test, p < 0,05).

Due to the previously observed biofilm inhibitory activity of Aii20J, the possible influence the AHL-lactonase on the bacterial diversity of the biofilms was further studied using the saliva from the same healthy patient used in previous experiments (Fig. 4 and Supplementary material Figs. 5, 6, 7 and 8) and from a periodontal patient. The inhibitory effect of the AHL-lactonase on *in vitro* oral biofilms formed by saliva samples was confirmed using different culture conditions (Fig. 5). A significant inhibition of the biofilms by Aii20J was observed when the samples from the healthy patient were inoculated in both BHI (50.08%), and BHI supplemented with 0.1% sucrose (45.60%) under aerobic conditions (Fig. 5a). The presence of the lactonase also reduced biofilm production at 24 h in anaerobic conditions (35.40%) (Fig. 5a). On the contrary, when using the crystal violet staining method in the periodontal sample, the inhibitory effect caused by Aii20J could only be observed in biofilms grown in BHI supplemented with 0.1% sucrose (BHIs) in aerobic conditions after 24 h (Fig. 5b). Though the crystal violet staining could not detect differences in some cases, the addition of Aii20J caused an apparent reduction in the amount of biofilm biomass that was harvested for the metagenomic analysis using samples of both, healthy and periodontal patients in the Amsterdam Active Attachment system after 24 h. The difference in biomass could be observed with the naked eye (Supplementary material Fig. 9).

**Figure 6:**
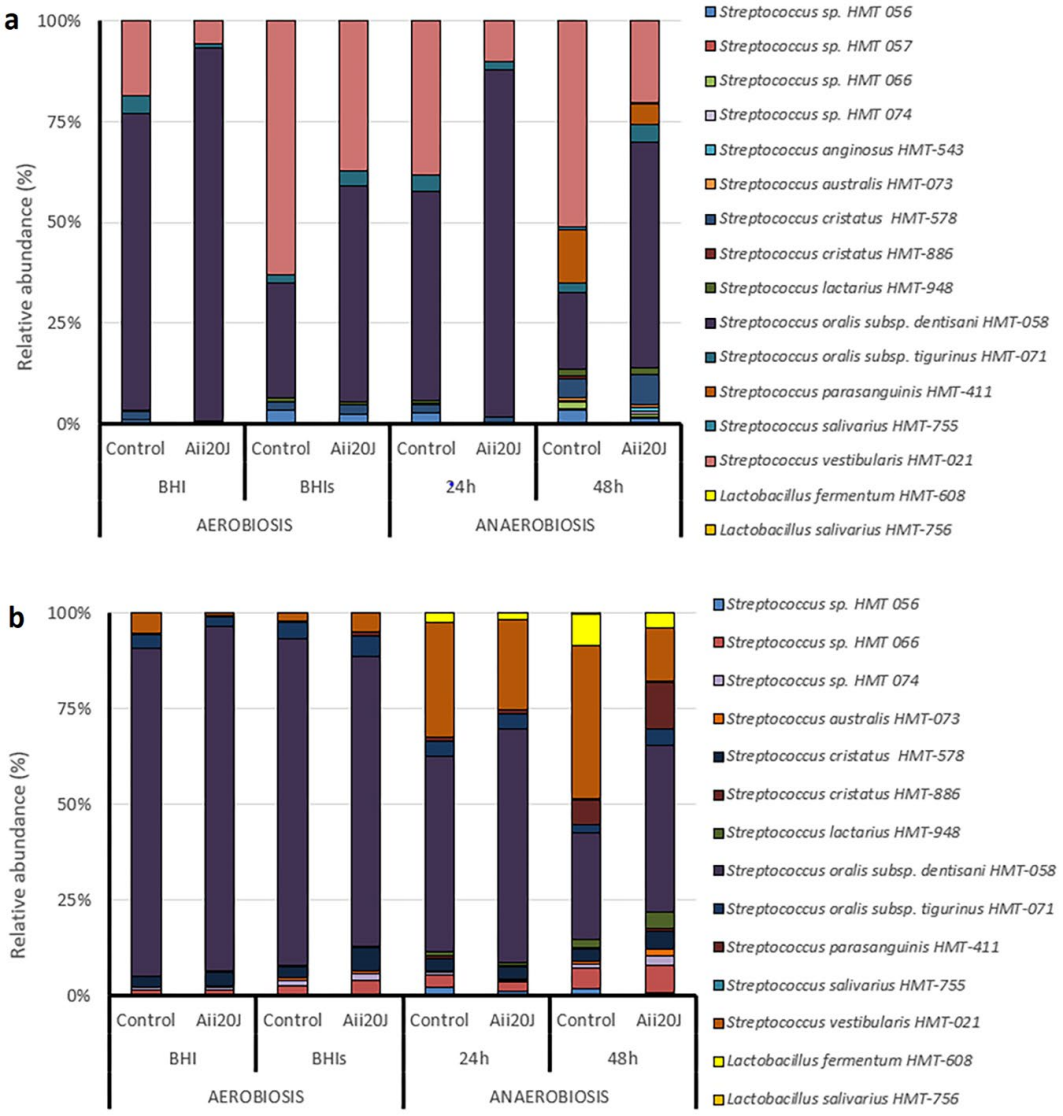
Bacterial diversity of the *in vitro* oral biofilms obtained from saliva samples from a healthy (**a**) and a periodontal (**b**) donor with and without the AHL-lactonase Aii20J (20 µg/mL). The cultures were incubated in aerobic conditions using BHI and BHI supplemented with 0.1% sucrose (BHIs) for 24 h and in aerobic conditions for 24 and 48 h using BHI.

The 16S rRNA gene sequences of DNA samples obtained from the biofilms grown in the different culture conditions were pyrosequenced with Illumina MiSec in order to evaluate the effect of the enzyme Aii20J on bacterial diversity. All retrieved sequences belonged to Firmicutes, especially to genus *Streptococcus*. Further experiments in which the collection of samples and sequencing method were optimized also revealed that 99.74% of the obtained sequences belonged to the genus *Streptococcus* with the selected culture conditions (BHI and BHI supplemented with 0.1% sucrose). Surprisingly, for biofilms dominated by Gram-positive species, both quantitative and qualitative changes were observed in bacterial diversity when the AHL-lactonase Aii20J was added (Fig. 6). The analysis of the relative abundance in the biofilms from the healthy donor revealed that Aii20J caused a clear and high increase of an OTU close to *S. oralis* subsp. *dentisani* and a concomitant decrease of a member of the *S. vestibularis* group in all tested conditions (Fig. 6a). This rise in the relative abundance of *S. oralis* subsp. *dentisani* was higher in anaerobic (34.27–37.08%) than in aerobic conditions (18.2–25.24%). Despite of the crystal violet stain could not reveal significant quantitative changes in the enzyme-treated biofilms obtained with the periodontal saliva (Fig. 5), an increase of *S. oralis* subsp. d*entisani* and a decrease of *S. vestibularis* were also observed, although these changes were lower than in the biofilms obtained from the healthy donor (Fig. 6b).

Furthermore, in the periodontal patient, an OTU close to *S. oralis* subsp. d*entisani* increased slightly with the enzyme when the biofilm was grown in BHI (85.81% versus 89.89%) but decreased if the culture media was supplemented with sucrose (84.50% versus 74.88%) (Fig. 6b). Moreover, Aii20J caused an apparent reduction of the *Lactobacillus* abundance when the samples from the periodontal donor were incubated in anaerobic conditions (Fig. 6b)

**Figure 7:**
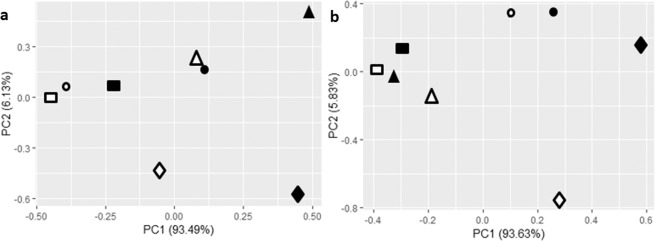
Principal components analysis of the species composition of *in vitro* oral biofilms obtained from healthy (**a**) and periodontal (**b**) saliva samples with and without the QQ enzyme Aii20J. Biofilms were cultured in BHI or BHI supplemented with 0.1% sucrose under aerobic and in BHI during 24 h and 48 h under anaerobic conditions. Closed symbols represent the control biofilms while open symbols represent the biofilms treated with the QQ enzyme Aii20J. Squares = BHI aerobic conditions, circles = BHI anaerobic conditions, triangles = BHI supplemented with 0.1% sucrose in aerobic conditions and diamonds = 48 h BHI in anaerobic conditions in both panels.

The effect of Aii20J on the bacterial composition of *in vitro* oral biofilms was also evaluated using PCA analysis (Fig. 7). PC1 explains the 93.49% and the 93.63% in the variance obtained from the healthy (Fig. 7a) and the periodontal (Fig. 7b) biofilms, respectively. Furthermore, in the samples obtained from the healthy patient, PC1 differentiates the treated biofilms from their respective untreated biofilms and is represented most notably by a differential abundance of members close to *S. oralis* subsp. *dentisani* and *S. vestibularis*. However, in the periodontal biofilms, PC1 allows for the differentiation of the biofilms depending on the aerobic or anaerobic conditions due to the higher microbial diversity obtained in anaerobic conditions for the periodontal donor.

### Effect of Aii20J on multi-species biofilms formed by oral pathogens

**Figure 8:**
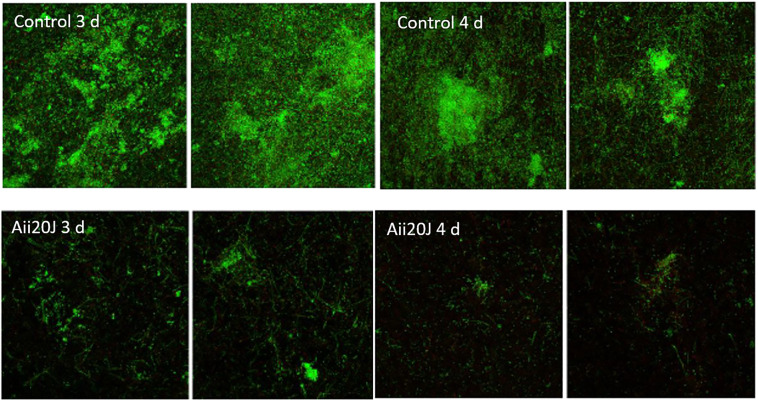
Confocal microscopy visualization (63×) of the effect of the QQ enzyme Aii20J (20 µg/mL) on multi-species biofilms formed by the oral pathogens *A. naeslundii, A. actinomycetemcomitans*, *F. nucleatum, P. gingivalis S. oralis* and *V. parvula* using fluorescence dyes Syto9 and propidium iodide. The biofilms were cultured in BHI-2 during 3 and 4 days in anaerobic conditions at 37 °C.

The effect of Aii20J (20 µg/mL) on *in vitro* mixed-species biofilms formed by six oral pathogens^46^ was checked using confocal microscopy in order to corroborate the inhibition of the saliva biofilms observed with the crystal violet staining method. When the oral pathogens *A. naeslundii, A. actinomycetemcomitans*, *F. nucleatum, P. gingivalis, S. oralis* and *V. parvula* were co-cultured during 3 or 4 days, an evident inhibition of biofilm formation was observed when Aii20J was added to the culture media (Fig. 8). The total area, as well as the area of live cells, were statistically higher in control biofilms than in the treated biofilms. The highest covered area was occupied by the control biofilms at day 3 and day 4 (870.70 ± 404.92 and 1,553.30 ± 1,091.14 µm^2^) in comparison to the treated biofilm (585.37 ± 379.57 and 546.68 ± 340.79 µm^2^). In the same way, the volume of live cells was statistically lower in the treated biofilms in comparison to the control biofilms. On the contrary, the thickness of the treated biofilms in comparison to control biofilms was similar on day 3 (31.97 ± 6.58 versus 33.41 ± 9.72 µm) but higher on day 4 (17.43 ± 5.69 versus 26.61 ± 7.74 µm). The porosity of these biofilms was also much higher than the control biofilms, indicating important differences in the structure of both types of biofilm.

## Discussion

Confirming previous studies that have already demonstrated the presence of AHL-type QS signals in saliva and sputum samples^17–20^, in this study we report the presence of this type of signal molecule in different oral samples. Moreover, the analysis of *in vitro* multi-species oral biofilms formed by *S. oralis, V. parvula, A. naeslundii, F. nucleatum, A. actinomycetemcomitans* and *P. gingivalis*, and of *P. gingivalis* axenic cultures revealed the presence of the QS signal OC8-HSL in small amounts. The current accepted paradigm does not assign a significant role to AHLs in dental plaque formation since these signals are almost completely excluded from the literature reviews on QS mechanisms in oral biofilms^13–16^. Although a few strains with the ability to produce AHLs were isolated from the human tongue surface and dental plaque samples^21–25^, the fact that AHLs could not be detected in pure cultures of pathogenic oral bacteria in previous studies^10–12^ is the reason why a minor role is attributed to these signals in the oral cavity. The low sensitivity of the biosensors may be partially responsible for the low number of AHL-producing strains isolated from the oral cavity. A high-sensitivity method such as mass spectrometry analysis is needed for the unequivocal detection of AHLs produced by bacterial strains. Moreover, the analyses should be performed using static cultures that favour biofilm formation and AHL production and the culture medium can also strongly affect AHL production. Since surface adherence or cell-to-cell adherence is required to activate AHL synthesis in some bacteria^34,48^ and specific bacteria modify the gene expression in oral biofilms^4^, these biofilm-promoting cultivation conditions may have triggered the expression of QS-related genes not expressed in axenic cultures or under agitated conditions. Moreover, AHLs may be only produced in mixed cultures, as demonstrated by the increase in AHL production in mixed cultures of *P. gingivalis*. In this sense, some bacteria are known to depend on the QS signals produced by others; for instance, *S. gordonii* requires AI-2 in order to form mixed biofilms with *P. gingivalis*^49^.

The most common AHL present in the extracted teeth and saliva samples was C8-HSL, which was found in all tested samples, with only one exception. C8-HSL and OC8-HSL were also the main AHLs found in saliva samples of a healthy donor used to generate different oral biofilm models, although some variability was found over time^19^. All patients who presented C8, C14 and C18-HSL in their saliva suffer from dental cavities while only C8 and occasionally C14-HSL could be found in those with periodontal disease. The higher diversity of AHL signals found in dental plaque from patients with caries is compatible with the lower pH that is expected in these samples since AHLs are more stable in acidic pH^50^. Even though all samples were acidified in order to allow the recovery of the lactone rings before analysis, the neutral or basic pH values in healthy dental plaque may have allowed a faster degradation of the open lactone-ring AHLs by other bacteria. Although these data seem to indicate a correlation between AHL profile and specific oral pathologies, the data obtained in the present study are not enough to establish an association between the observed oral pathologies and the amount and/or type of AHL detected.

In the present work, the production of a small amount of OC8-HSL (0.30 ng/mL) by *P. gingivalis w*as detected for the first time. The most common AHL-dependent QS system is based on two proteins: a LuxI-type synthase and a LuxR-type receptor^51^, although other AHL synthases belonging to the families LuxM/AinS and HdtS have been described^52,53^. An ORF with 25% identity and 48% amino acid similarity with the AHL-synthase HdtS was identified in *P. gingivalis* W83^26^ indicating the presence of at least a putative AHL-synthase in this species. Additionally, a LuxR homologue, designated as CdhR (Community Development and Hemin Regulator), that controls the transcription of the *hmu* operon responsible for iron/hemin uptake was found in this bacterium^27,28^. Furthermore, a recent study has identified a non-classical QS synthase in an AHL-producing *Psychrobacter* strain, indicating the possible existence of yet unknown AHL-synthases not homologous to those described so far^54^. Therefore, the presence of other unknown AHL-synthases or receptors in *P. gingivalis* cannot be disregarded. The influence of different AHLs, as well as AHL-analogues on *P. gingivalis*, were evaluated in previous works showing that when C14-HSL was externally added, changes in both protein expression and bacterial growth were observed^26,29^, indicating the presence of an AHL receptor. Furthermore, in a previous work, we demonstrated that the exogenous addition of specific AHLs generated changes, not only in the bacterial composition but also in metabolic activity of *in vitro* oral biofilms obtained from saliva^19^. C14 and C18-HSL (both AHLs found in oral samples in the present work) reduced the production of lactic acid in cariogenic and commensal *in vitro* saliva biofilms, respectively. On the contrary, the addition of C8-HSL, the major AHL found in the same samples, caused a significant decrease in the production of lactate in both types of biofilm. Additionally, C18-HSL also produced an increase in the protease activity of the commensal biofilms^19^. These effects may be patient-specific, and therefore further studies are needed to establish the role of these signals in the development of cariogenic dental plaque. Furthermore, the addition of C6-HSL increased the relative abundance of *Peptostreptococcus* and *Prevotella*, resulting in a shift towards a periodontal bacterial composition profile^19^. All these data indicate that AHLs perform a critical role in oral biofilm formation, even in those dominated by Gram-positives. In another study^55^ the cariogenic potential of the dental plaque was reduced by the addition of a C12-HSL analogue, which induced changes in the relative abundance of the biofilm bacterial composition compared to the control, decreasing *Streptococcus* spp. (from 61% to 33%) and increasing *Actinobacillus* spp. (from 5% to 29%). Nevertheless, this effect could be derived from growth inhibition, since the toxicity of OC12-HSL and its tetramic acid degradation product has been reported for Gram-positive bacteria^56^, and no competition was observed between the analogue and the natural AHL.

The importance of AHL-mediated QS systems in the oral ecosystem is further supported by a high prevalence of QQ activity against short-chain AHLs among cultivable bacteria isolated from dental plaque and saliva obtained from healthy and periodontal donors (14–47%). These percentages may be even higher considering that in environmental samples, QQ activity against short-chain AHLs is less frequent than against long-chain AHLs^46,57^. These results of QQ prevalence among the cultivable oral strains are similar to those obtained in marine samples^43,57–61^ which is the environment with the highest QQ activity described thus far. The frequency of QQ strains was significantly higher in dental plaque samples from a periodontal patient, indicating the potential importance of QQ activity in the establishment of a dysbiosis that should be further investigated. Additionally, the capability of saliva from several donors to interfere with C12-HSL was observed. An important antimicrobial activity was also found among the isolates with 73 of them being able to inhibit the growth of the *A. tumefaciens* biosensor. Antimicrobial activity was more abundant in the healthy patient (60.27%) than in the periodontal one (39.72%). This higher abundance of strains with antimicrobial activity in the healthy patient could also be related to the health status of the donor. Recently, the use of the probiotic strain *Streptococcus dentisani* (reclassified as *S. oralis subsp. dentisani*) was proposed for the control of oral diseases since it is present in a high percentage of healthy patients and can inhibit the growth of the primary oral pathogens due to bacteriocin production and pH buffering of the oral cavity^62,63^.

Though several works have reported the potential use of AI-2 inhibitors in reducing dental plaque^35,64–68^, to the best of our knowledge, this is the first study that demonstrates that an AHL-lactonase inhibits oral biofilm formation, strongly supporting the critical role this type of QS signal plays in oral bacterial communities. The addition of the wide-spectrum AHL-lactonase Aii20J also affected the 6-species biofilms composed of the odontopathogens *A. naeslundii, A. actinomycetemcomitans, F. nucleatum, P. gingivalis, S. oralis* and *V. parvula*. The thickness, as well as the porosity, increased but the covered area and the volume of live cells were lower when the biofilms were treated with the lactonase Aii20J. The biofilm inhibitory activity of Aii20J was confirmed in biofilms generated using different saliva samples from healthy and diseased patients using the xCELLigence system (25.15–93.4%). Furthermore, the effect of Aii20J on *in vitro* oral biofilms was evaluated in different culture conditions using the Active Attachment system on glass coverslips and the crystal violet staining assay, showing a significant inhibition of biofilms obtained with saliva samples from a healthy patient when cultivated in both aerobic and anaerobic conditions. The analysis of the relative abundance and PCA analysis revealed that the presence of Aii20J caused a high increase of *S. oralis* subsp. *dentisani* and an important decrease of *S. vestibularis* in the biofilm formed by saliva obtained from a healthy patient. Although the AHL-lactonase did not cause an inhibition in the quantity of biofilm formed by a periodontal saliva sample as measured using the crystal violet staining assay, a decrease of the biofilm biomass could be observed with the naked eye when the biofilms were harvested for metagenomic analysis, indicating a low sensitivity of the crystal violet stain^35,69^. A shift in the bacterial diversity similar to that observed in the healthy saliva sample was also observed. This effect of Aii20J on the bacterial composition of the oral biofilms could be used as a novel strategy in healthcare through the promotion of the antimicrobial activity present in some commensal oral bacteria such as *S. oralis* which can inhibit the growth of several oral pathogens such as *A. actinomycetemcomitans, P. gingivalis* and *P. intermedia*^70^.

The important inhibition observed in mixed *in vitro* biofilms, which are composed mostly by Gram-positive species (99.9%), when treated with Aii20J could be explained either by the possible interaction between the AHL and Gram-positive bacteria or by interactions with minority Gram-negative components of the oral biofilm. Some bacteria do not produce AHLs but possess LuxR homologs. These LuxR orphans interact with the QS molecules present in the environment produced by other microorganisms^71^. A gene belonging to LuxR-family of regulatory proteins was predicted in the genome of *S. mutans*, suggesting the existence of a LuxR orphan^72^. An important Gram-positive pathogen, *Staphylococcus aureus*, responds to the OC12-HSL produced by *Pseudomonas aeruginosa* in a saturable and specific manner, resulting in the inhibition of the production of exotoxins and the enhanced expression of the protein A, an important surface protein involved in several virulence mechanisms^73^. Furthermore, since the production of AHLs has been reported in several Gram-positive bacteria belonging to Actinobacteria and Firmicutes phylum^74–77^ as well as in bacteria that do not possess a known AHL synthase^54^ we cannot exclude that completely unknown AHL synthases are present in oral pathogenic bacteria. In addition, LuxR-type receptors can be activated by several signalling molecules besides acyl-HSLs produced by LuxI homologues, regarded as “dialect” synthases^78^. Hence, all these data indicate that the QS network in the oral cavity may be much more complicated than the currently accepted model in which oral bacterial communication is only mediated by AIPs and AI-2, meanwhile the AHLs’ signalling role is considered of minor relevance.

The observation of the presence of AHL-type QS signals in oral samples as well as in *in vitro* oral biofilms and the high abundance of strains with QQ activity strongly indicates that AHL-mediated QS systems play an important role in the oral cavity. This conclusion is further supported by previous experiments that demonstrated the important effects of the exogenous addition of AHLs to different biofilm models^19^. The significant quantitative and qualitative effects of the AHL-lactonase Aii20J on saliva and mixed oral pathogen biofilms confirms this critical role and opens new possibilities in the prevention and treatment of oral diseases. Further analysis is necessary to confirm and complete our knowledge regarding the role and relevance of AHL-mediated processes in the maintenance of the ecological equilibrium of the microbial inhabitants of the oral cavity through competitive and cooperative interactions, as well as on the possibility of applying QQ strategies for the control of oral diseases.

## Methods

### Bacterial strains and culture media used

The pure cultures of *P. gingivalis* ATCC33277 and co-culture of this bacterium with *S. oralis* CECT907T or *S. gordonnii* ATCC49818 were performed in BHI-2 [BHI supplemented with 2.5 g/L mucin, 1 g/L yeast extract, 0.1 g/L cysteine, 2 g/L sodium bicarbonate, 5 mg/L hemin, 1 mg/L menadione and 0.25% (v/v) glutamic acid] in anaerobic conditions for 16 h at 37 °C. Multi-species biofilms formed on hydroxyapatite discs (HA) by the six oral pathogens *S. oralis* CECT907T*, V. parvula* NCTC11810*, A. naeslundii* ATCC19039*, F. nucleatum* DMSZ20482*, A. actinomycetemcomitans* DSMZ8324 and *P. gingivalis* ATCC33277 were cultured in BHI-2 with or without the Aii20J (20 µg/mL) in anaerobic conditions for 4 days at 37 °C. The planktonic growth was performed in the absence of HA discs in the microtiter wells. After incubation, the culture media was acidified (pH 2) and stored at 4 °C until its use.

The *in vitro* oral biofilms obtained from saliva samples were cultured in Brain Heart Infusion (BHI) broth (Cultimed) or BHI supplemented with 0.1% sucrose (BHIs) with or without the Aii20J (20 µg/mL) in aerobic conditions and BHI and BHI-2 in anaerobic conditions at 37 °C.

Saliva samples and dental plaque samples were collected from a healthy and a periodontal donor for bacterial isolation and functional screening. The dental plaque samples were introduced in tubes with thioglycollate medium and vigorously vortexed for homogenization. Four series of 10-fold dilutions (10^−1^, 10^−2^, 10^−3^ and 10^−4^) of saliva samples and homogenated dental plaque samples were prepared in thioglycollate medium for each sample and plated in Columbia agar (Scharlau) and Schaedler agar with blood (Scharlau). Plates were incubated at 37 °C for 15 days using anaerobic jars GENBox (Biomerieux). A total of 567 strains, 287 isolates from dental plaque and 280 isolates from saliva, were randomly picked up and isolated to be used for QQ functional screening.

### Samples collection and growth conditions

All the saliva samples, dental plaque samples and extracted teeth were obtained from volunteers after signing an informed consent approved by the Comité Autonómico de Ética de la Investigación de Galicia (protocol 2009/319 modified in July 2017). The extracted teeth were introduced in tubes with PBS pH 2 and stored at 4 °C until analysis. The patients were asked to spit, and the saliva samples were collected in sterile tubes, diluted in PBS 6.5 and use as inoculums immediately or diluted in PBS pH 2 and stored at 4 °C until their use.

The study protocol has received ethical approval from Comité Autonómico de Ética de la Investigación de Galicia (protocol 2009/319 modified in July 2017) formed by Manuel Portela, Irene Zarra, Paula López, Juan Vázquez, Jesús Alberdi, Rosendo Bugarín, Juan Casariego, Xoán Casa, Juana Cruz, Juan Cueva, José A. Fernández, José L. Fernández, José Ferreira, Pablo Nimo, Pilar Gayoso, Agustín Pía, Salvador Pita, Carmen Rodríguez-Tenreiro, Susana Romero and Asunción Verdejo. All methods were performed in accordance with relevant guidelines and regulations.

### Extraction and identification of AHLs by HPLC-MS

Remaining AHLs in saliva samples, extracted teeth and acidified supernatants from the *in vitro* oral biofilms were extracted twice into an appropriate volume of dichloromethane and ethyl acetate. Then, the solvents were evaporated to dryness at 40 °C. AHLs present in the samples were reconstituted in an appropriate volume of acetonitrile and quantified by HPLC-MS methodology as previously described^19^. Pure AHLs covering the whole range of AHL lengths (from C4-HSL to C18-HSL), both C3 hydroxy- or oxo- substituted and un-substituted were obtained from Sigma-Aldrich and the University of Nottingham and used as an external standard for quantification.

### Quorum sensing activity assay

The 568 strains of the cultivable oral collection were screened for their capability to activate the AHL biosensor *A. tumefaciens* NTL4 in a previous work^46^. Strains were cultured in microtiter plates in 200 μL of Schaedler supplemented with vitamin K for 48 h under anaerobic conditions at 37 °C. Plates were centrifuged, and the supernatants were transferred to a new plate. The presence of AHLs after the incubation period was detected by adding 50 μL of a mixture of soft AB medium^79^ (0.2% agar) with 5-bromo-4-chloro-3-indolyl-β-D-galactopyroside (X-GAL, 80 µg/mL) and an overnight culture of *A. tumefaciens* NTL4 (1:5) on top of the supernatants in microtiter wells. The plates were incubated for 6–8 h at 30 °C and the production of blue colour on the surface of the wells was checked. AB medium pH 6.5 plus the C6-HSL (10 μM) was used as control. *A. tumefaciens* NTL4 was cultured at 22° in Luria-Bertani (LB), or AB medium supplemented with 30 µg gentamycin/mL.

### Quorum quenching activity assay

The QQ activity of the 568 cultivable oral strains was tested using solid microtiter plate assays^46^ carried out with the AHL biosensors *Chromobacterium violaceum* CV026^80^ for C6-HSL. 200 μL of 48 h cultures carried out in microtiter plates using Schaedler supplemented with vitamin K under anaerobic conditions were centrifuged and pellets were washed with phosphate-buffered saline (PBS) pH 6.5 and resuspended in another 200 μL of the same buffer in order to avoid lactonolysis of the exogenous AHLs by high pH values^50^. These cell suspensions were used for a live-cell AHL degradation assay, by adding C6-HSL (10 µM) and incubating for 24 h at 22 °C. After the incubation period, the presence of AHLs was detected by adding 50 μL of a mixture of soft Luria-Bertani (LB) (0.2% agar) and an overnight culture of the *C. violaceum* biosensor on top of the cell suspension in microtiter wells. The plates were incubated for 24 h at 30 °C, and the production of violacein was observed. PBS pH 6.5 plus C6-HSL AHL (10 μM) was used as control.

### Production and purification of the AHL-lactonase Aii20J

The expression of Aii20J was performed as previously described^33,34^. Briefly, the *E. coli* BL21(DE3) plysS strain expressing the recombinant protein was inoculated into fresh LB medium with kanamycin (25 µg/mL) at 37 °C. The protein expression was induced when the culture reached 0.6 O.D. by the addition of 0.1 M Isopropyl-D-thiogalatopyranoside (IPTG) followed by further incubation of 5 h. Then, the culture was centrifuged, and the pellets were resuspended with 20 mL of PBS buffer, lysed by sonication on ice and centrifuged again. Aii20J was purified using the His GraviTrap affinity column (GE Healthcare) protein purification kit.

### Biofilm measurement and analysis

The *in vitro* oral biofilms were first measured using the xCELLigence System RTCA SP (ACEA, Biosciences Inc.)^35^. E-plates 16 (ACEA, Biosciences Inc.) were inoculated with 80 μL of undiluted saliva, 100 μL of BHI or BHI supplemented with 0.1% sucrose and with or without 20 μL of the AHL-lactonase Aii20J (final concentration 20 µg/mL). 20 μL of PBS pH 6.5 instead of the lactonase were added to the control wells. The system was incubated at 37 °C for 24 h.

The *in vitro* oral biofilms were also cultured using a modification of the Amsterdam Active Attachment model^19,45^ assembled with glass coverslips (18×18 mm). All biofilms were inoculated in 12-well using a 1:50 dilution of saliva in the different culture media. Plates were incubated at 37 °C in aerobic or anaerobic conditions. After the first 12 h culture change, the media were refreshed every 12 h for aerobic conditions and every 24 h for anaerobic conditions. The AHL-lactonase Aii20J (final concentration 20 µg/mL) was added with every medium exchange, including the initial 12 h inoculation step. After incubation at 37 °C, the supernatants were removed, and the wells were rinsed with distillate water. When the biofilms were dried, 0.04% Crystal Violet (Gram-Hucker, Panreac) solution was added to all wells, and after 20 minutes, the excess of dye was removed by washing several times. Bound crystal violet was released by adding 33% acetic acid. The absorbance was measured at 590 nm^35^.

### Microbial DNA extraction

Metagenomic DNA extraction was done using “DNeasy PowerBiofilm Kit” (Qiagen) following the manufacturer’s instructions. DNA concentration was measured using UV-Vis Spectrophotometer Q5000 (Quawell).

### Library preparation

Microbial genomic DNA (2.5 µL) was used to amplify the 16 rDNA using the primers Bakt_341F and Bakt_805R^81^. After size verification, the library was sequenced using a MiSeq PE300 Sequencer (Illumina) following the manufacturer’s instructions.

### Bioinformatics and microbial diversity analysis

Quality assessment and preprocessing data were performed using FastQC, FLASH^82^, CUTADAPT 1.3^83^. The sequences were labelled using Qiime^84^ and processed using VSERACH. The taxonomic affiliations were obtained using Qiime and the database SILVA^85^.

The principal component analysis (PCA) was performed in RStatistics^86^ doing the graphs with ggfortify and ggplot2^87^.

### Microscopic visualization and analysis

Multi-species biofilms formed by the different oral pathogens were cultured with or without the Aii20J (20 µg/mL) in anaerobic conditions for 4 days at 37 °C. After incubation, the supernatant was removed. The HA discs were immersed twice in PBS to remove the planktonic cells, and the biofilms were stained using the LIVE & DEAD Baclight Bacterial Viability Kit, L7012 (Molecular Probes, Eugene, OR, USA) as previously described^88^. HA discs were immersed in equal volumes of SYTO9 (0.02 mM) dye and propidium iodide (PI) (0.12 mM) dye diluted in PBS, and the mixture was incubated for 10 minutes at room temperature, avoiding exposure to light. Biofilms were rinsed once with phosphate-buffered saline (PBS) and were examined under 63x magnification, using a Leica TCS SP5 confocal laser scanning microscope (Leica Microsystems, Heidelberg, Germany). For each condition, four biofilms and four fields per biofilm were observed. The biofilm variables studied were quantified using MetaMorph v1.5 software (Molecular Devices, LLC, Sunnyvale, USA) as previously^87^.

### Statistical analyses

The Student’s t-test test was performed to determine the statistical significance of the differences in cell index and optical density between the control and the treated-wells. Differences in area and volume of live and dead cells in the multi-species oral biofilms between negative control and Aii20J Lactonase treated biofilms were assessed with the Mann-Whitney U test. Significant differences were determined at p < 0.05.

## Supplementary information

Supplementary Information.
