# Supplementary material for: Acyl homoserine lactone-mediated quorum sensing in the oral cavity: a paradigm revisited
Source: Sci Rep. 2020 Jun 17;10:9800. doi: 10.1038/s41598-020-66704-4 (PMC7300016; doi:10.1038/s41598-020-66704-4)
Supplement: Supplementary file 1 — Supplementary Information. [file 41598_2020_66704_MOESM1_ESM.docx]

**Acyl homoserine lactone-mediated quorum sensing in the oral cavity: a paradigm revisited**

Andrea Muras^1^, Paz Otero-Casal^2,3^, Vanessa Blanc^4^, Ana Otero^1^

Departamento de Microbioloxía e Parasitoloxía, Facultade de Bioloxía-CIBUS, Universidade de Santiago de Compostela, Santiago de Compostela, Spain^1^; Departamento de Ciruxía e Especialidade Médico-Cirúrxica, Facultade de Medicina e Odontoloxía, Universidade de Santiago de Compostela, Santiago de Compostela, Spain^2^; Unity of Oral Health, C.S. Santa Comba-Negreira, SERGAS, Spain^3^.Department of Microbiology, Dentaid Research Center, Dentaid S.L.,Barcelona, Spain^4^; #Corresponding author: anamaria.otero@usc.es


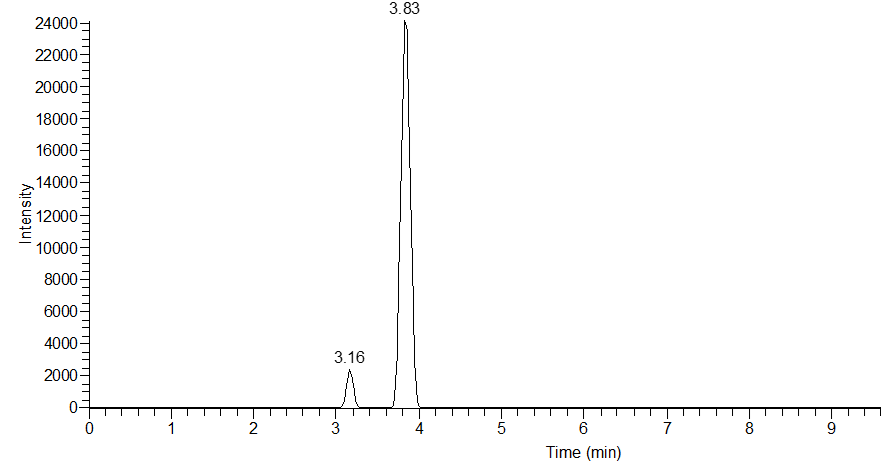

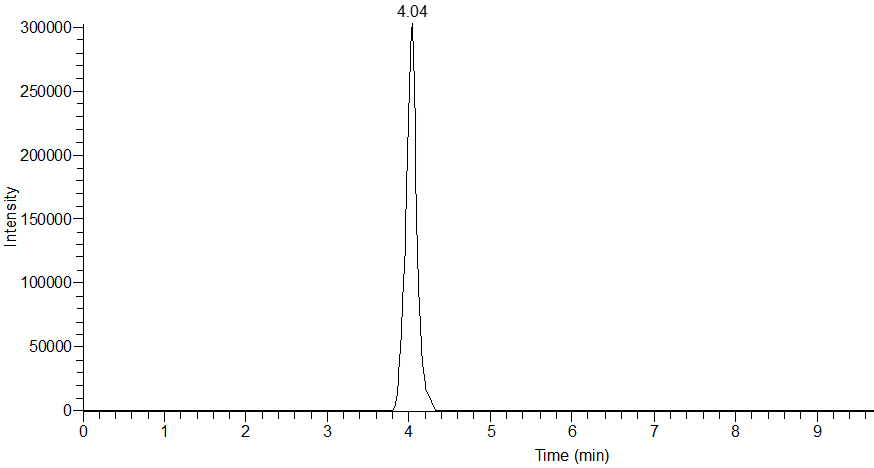


**Synthetic C8-HSL**

**Saliva C8-HSL**


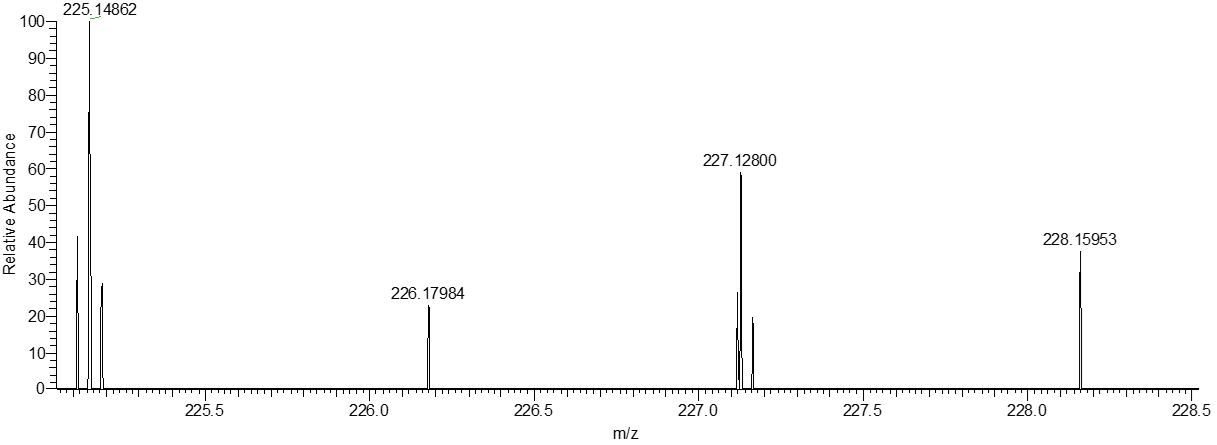


**Saliva C8-HSL**


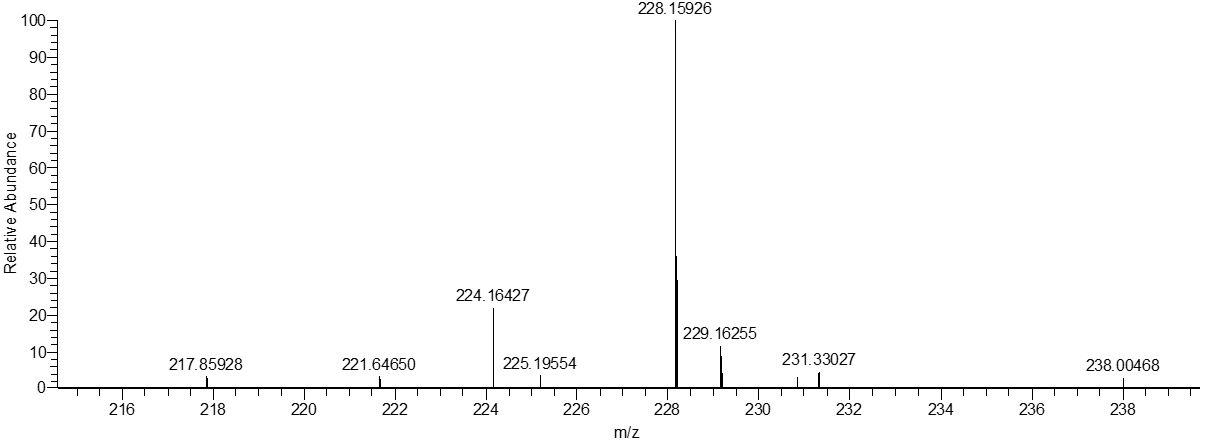


**Synthetic C8-HSL**

**Figure 1. Ion chromatograms from HPLC-MS analysis of C8-HSL with retention time and identification of peaks detected in saliva samples and synthetic standards.**


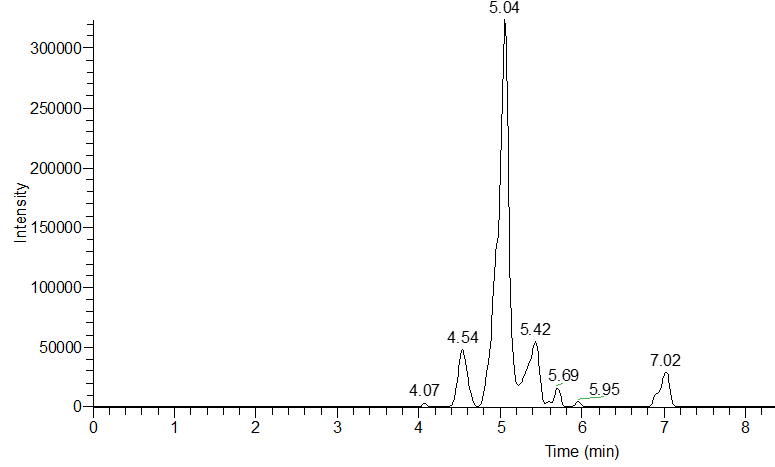

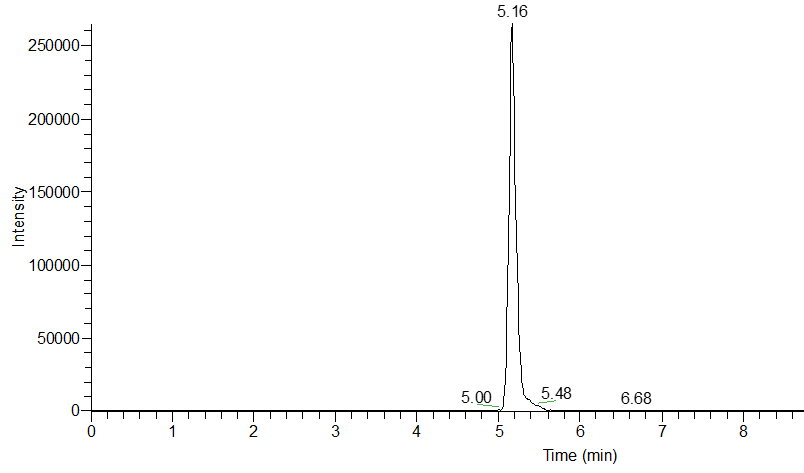


**Synthetic C14-HSL**

**Saliva C14-HSL**


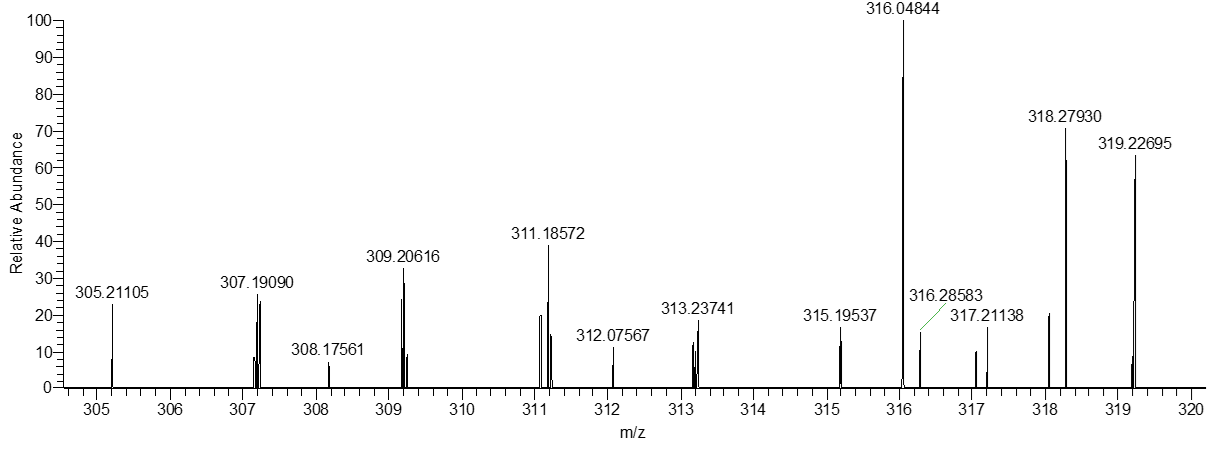


**Saliva C14-HSL**


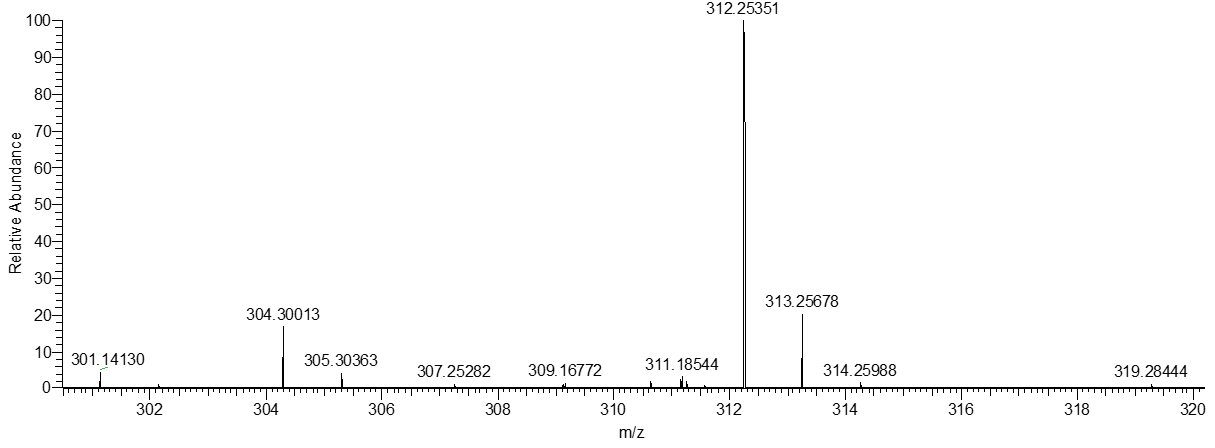


**Synthetic C14-HSL**

**14-HSL**

**Figure 2. Ion chromatograms from HPLC-MS analysis of C14-HSL with retention time and identification of peaks detected in saliva samples and synthetic standards.**


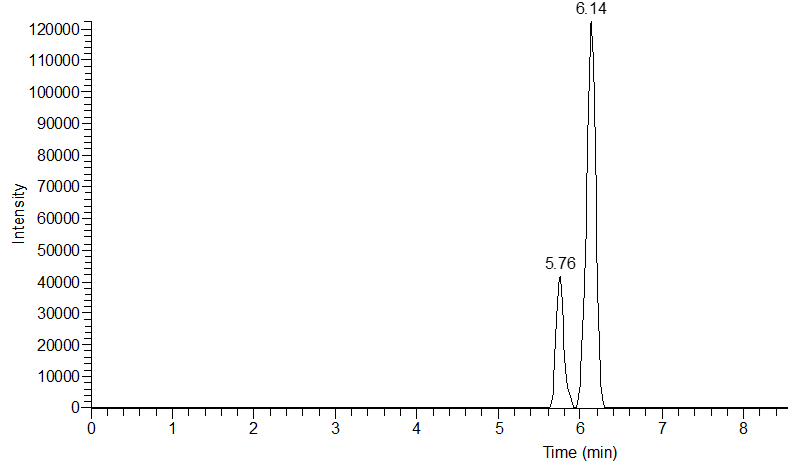


**Saliva C18-HSL**


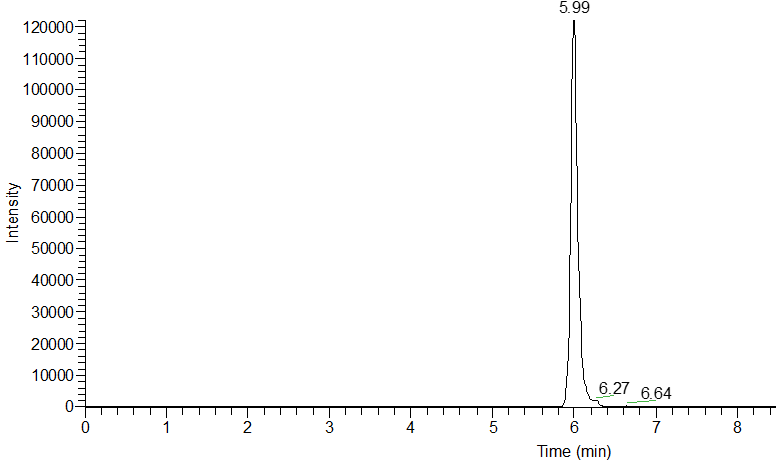


**Synthetic C18-HSL**

**14-HSL**


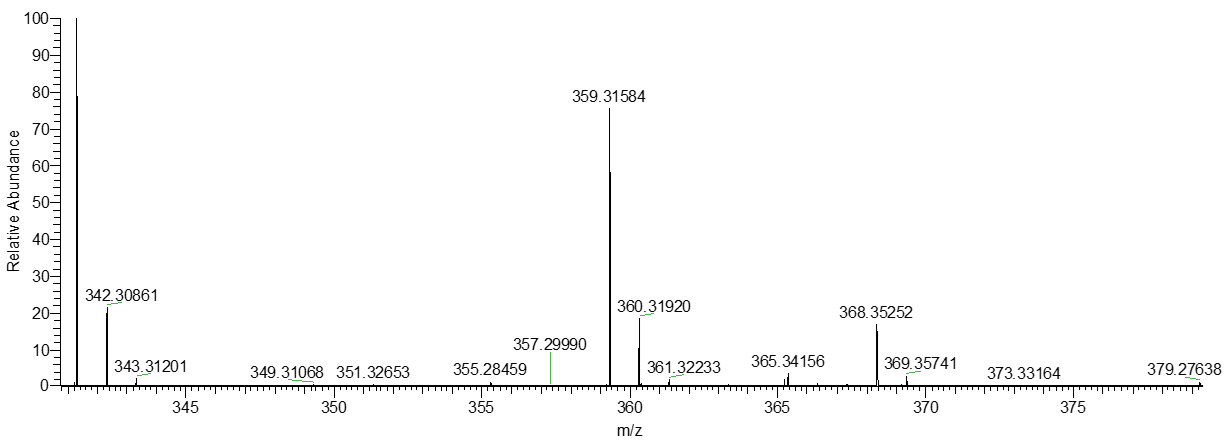


**Saliva C18-HSL**

**Synthetic C18-HSL**

**14-HSL**


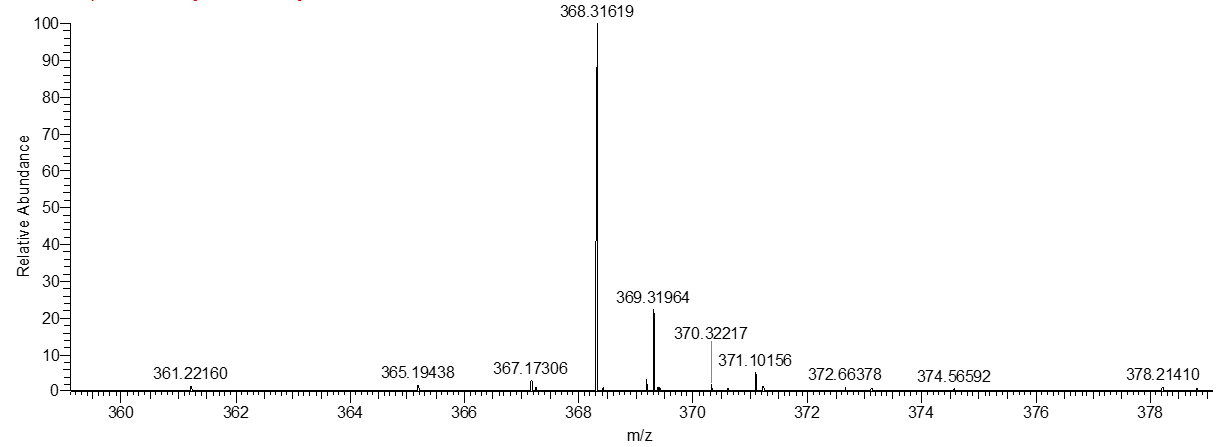


**Figure 3. Ion chromatograms from HPLC-MS analysis of C18-HSL with retention time and identification of peaks detected in saliva samples and synthetic standards.**

**Table 1. Total number and percentage of the isolated strains with quorum quenching activity against C6-HSL using the 96-well microtiter plates solid assay with *C. violaceum* CV026.**

|  | | Nº Isolates | QQ against  C6-HSL | %QQ against  C6-HSL |
| --- | --- | --- | --- | --- |
| HEALTHY | Saliva | 145 | 47 | 32.41 |
|  | Dental plaque | 150 | 21 | 14 |
| PERIODONTAL | Saliva | 135 | 38 | 28.14 |
|  | Dental plaque | 137 | 64 | 46.71 |

**Figure 4. Effect of the addition of the AHL-lactonase Aii20J (white circle) on oral biofilm formation (black circle) formed by a saliva sample obtained from unhealthy donor S6 in BHI (a) and BHI supplemented with 0.1% sucrose (b) measured using the xCELLigence® system. The figure shows how the inhibitory effect of the Aii20J lactonase cannot be observed when the cell index of the biofilm is lower than 0.15.**


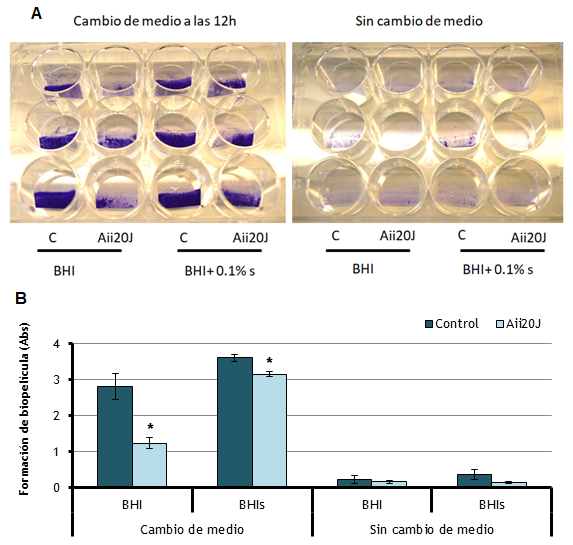


BHI

BHI

BHIs

BHIs

**a**

**Figure 5. Visual comparison (a) and biomass estimation (b) of *in vitro* oral biofilm formation with or without refreshment of the culture media at 12 h using the Active Attachment model stained with crystal violet (O.D. 590 nm). The biofilms were obtained from a saliva sample from a healthy donor and cultured using BHI and BHI supplemented with 0.1 % sucrose with and without AHL-lactonase Aii20J (20 µg/mL) in aerobic conditions for 24 h at 37C. The significant differences are indicated with an asterisk (Student's t-test, p<0.05) (n= 3).**


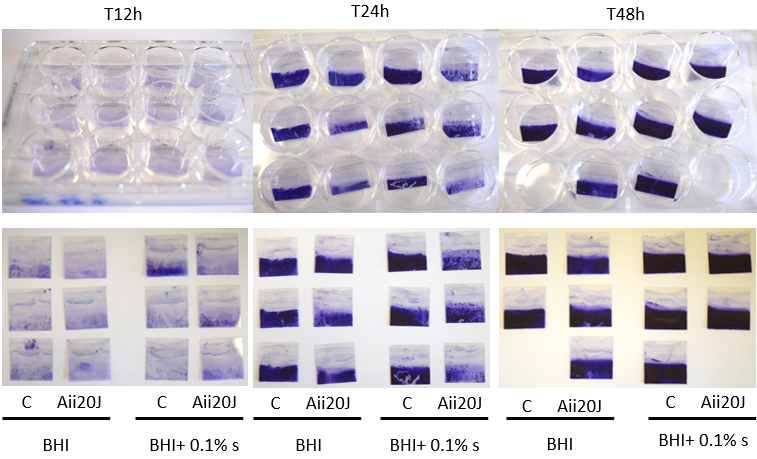


BHIs

BHI

BHIs

BHIs

BHI

BHI

**b**

**a**


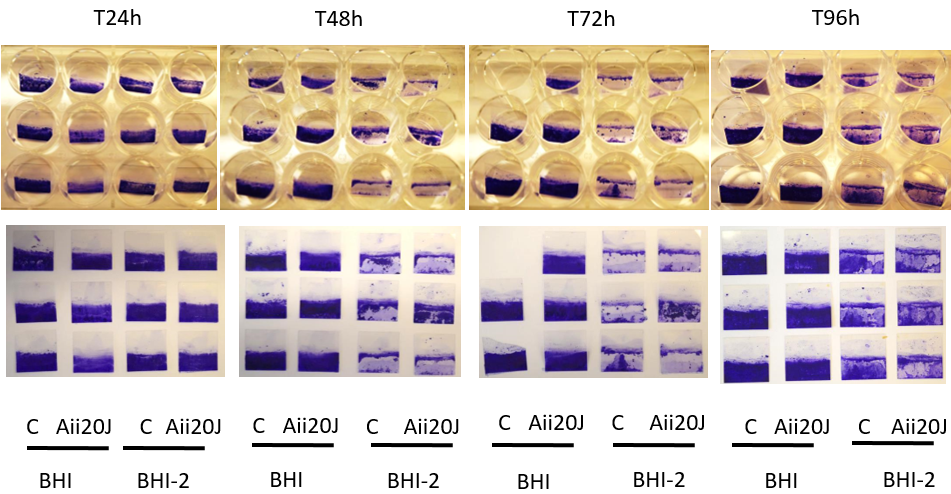


BHI-2

BHI-2

BHI

BHI

BHI-2

BHI

BHI-2

BHI

**Figure 6. Effect of the addition of the AHL-lactonase Aii20J (20 µg/mL) on *in vitro* oral biofilms obtained from a saliva sample from a healthy patient cultured aerobically (a) and anaerobically (b) at 37ºC using the Active Attachment model and measured with the crystal violet staining assay. The aerobic cultures (a) were done in BHI and BHI supplemented with 0.1% sucrose (BHIs) and sampled at 12 h, 24 h and 48 h. In anaerobic conditions (b) the cultures were done in BHI and BHI-2 for 24 h, 48 h, 72 h and 96 h.**

**Figure 7. Effect of untreated, autoclaved and enzyme-free filtrate of the AHL-lactonase Aii20J (20 µg/mL) on saliva biofilm formation using the Active Attachment system quantified using the crystal violet assay. The significant differences are indicated with an asterisk (Student's t-test, p<0.05) (n= 3).**

**Figure 8. Effect of the addition of the AHL-lactonase Aii20J (20 µg/mL) on *in vitro* oral biofilm formation with a saliva sample from a hyper-biofilm forming healthy patient (a) and saliva samples from other healthy and periodontal patients (b) using the Active Attachment model and measured with the crystal violet staining assay. The biofilms were incubated in aerobic conditions using BHI for 24 h at 37°C. The culture media was exchanged after 12 h. The significant differences are indicated with an asterisk (Student's t-test, p<0.05) (n= 3).**

**
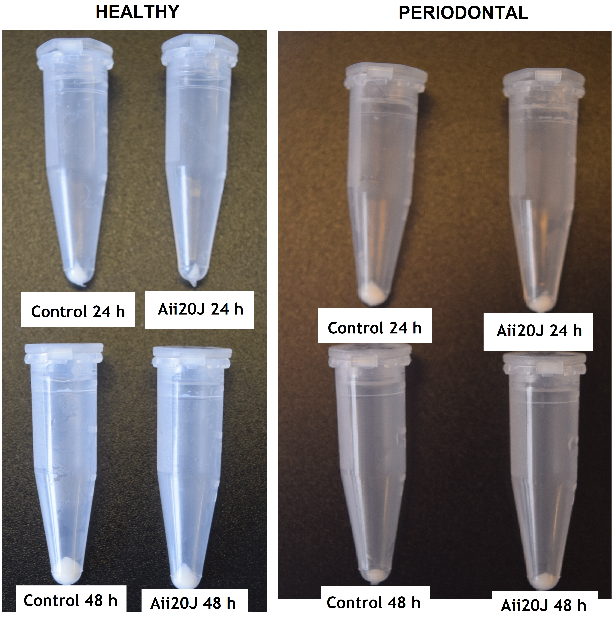
**

**Figure 9. Picture of the biofilm biomass collected using the Active Attachment model. Saliva samples from a healthy and a periodontal donor were cultured in BHI for 24 and 48 h in anaerobic conditions. The treated wells were supplemented with the AHL-lactonase Aii20J (20 µg/mL). Differences in the biofilm biomass can be observed that are not detected by the crystal violet staining method (Figure 5).**
